# The effect of facility-based nutrition education and counseling on dietary intake and supplemental iron folic acid use among pregnant women: a cluster randomised controlled trial

**DOI:** 10.1017/S1368980025101304

**Published:** 2025-11-03

**Authors:** Afrah Mohammedsanni, Demewoz Haile, Bilal Shikur Endris, Yirgu Gebrehiwot, Eskeziaw Abebe Kassahun, Seifu Hagos Gebreyesus

**Affiliations:** 1 Department of Nutrition and Dietetics, School of Public Health, College of Health Sciences, Addis Ababa University, Addis Ababa, Ethiopia; 2 Department of Obstetrics and Gynecology, School of Medicine, College of Health Sciences, Addis Ababa University, Addis Ababa, Ethiopia

**Keywords:** Nutrition counseling, ANC counseling, Nutrition education, Pregnancy, Dietary practice, Pregnant women

## Abstract

**Objective:**

Nutrition education plays a crucial role in improving the nutritional status of pregnant women, yet evidence of its impact in low-income settings like Ethiopia is limited. This study evaluated the effectiveness of facility-based nutrition education and counseling on pregnant women’s knowledge, dietary practices, and Fe-folic acid supplement use.

**Design:**

A cluster randomised controlled trial was conducted in Addis Ababa, Ethiopia, involving 683 pregnant women across twenty health centres assigned to intervention or control groups. Antenatal care providers in the intervention group received training on pregnancy nutrition and counseling, while the control group continued standard care per national guidelines. A total of 683 pregnant women were enrolled during their first and second antenatal care (ANC) visits. Mixed-effects linear regression was used to evaluate outcomes.

**Study setting:**

The study was conducted in Addis Ababa, Ethiopia, from August to December 2017.

**Participants:**

Pregnant women attending ANC follow-ups and healthcare providers working in ANC units.

**Results:**

The intervention group demonstrated significant improvements in knowledge, including iodised salt use (difference-in-differences (DID) 23 %), correct Fe-folic acid supplementation duration (DID 68 %) and the need for additional meals during pregnancy (DID 49·9 %). Dietary practices improved with higher dietary diversity (DID 32·3 %), increased dairy consumption (MD 1·2 *v*. –0·1; DID 1·2 per week) and higher Fe-folic acid supplementation (MD 4·9 *v*. 1·6; DID 3·2 per week).

**Conclusion:**

Nutrition education and counseling during ANC visits significantly improved pregnant women’s knowledge and dietary practices. Integrating and strengthening these interventions into routine ANC services could effectively enhance dietary intake and health outcomes.

Pregnancy is a critical phase in women’s lives, requiring a healthy diet rich in essential nutrients to ensure maternal and fetal well-being. The first 1000 d from conception to a child’s second birthday are particularly important for a healthy pregnancy, optimal fetal development and long-term health outcomes^(1)^. A pregnant woman’s diet plays a critical role in fetal development, pregnancy outcomes and her postpartum well-being by ensuring sufficient essential nutrients to support growth and reduce complications^(2)^.

Adequate dietary intake during pregnancy has positive fetal outcomes and health during childhood and beyond^(3,4)^. However, for many pregnant women, particularly in low- and middle-income countries, the dietary intake of vegetables, meat, dairy products and fruits are often insufficient to meet these needs^(5,6)^. Poor dietary intake lacking essential nutrients can lead to serious adverse perinatal outcomes such as intrauterine growth restriction, preterm birth, low birth weight and stillbirth^(7,8)^. In Ethiopia, maternal malnutrition affects 29·1 % of pregnant women and remains a major public health issue^(9)^. In 2019, 40 % of pregnant women did not take any Fe supplements, and only 11 % took Fe tablets for the recommended duration of 90 d or more^(10)^.

Nutrition interventions during pregnancy have received increasing attention in recent decades due to their critical role in enhancing maternal and fetal health outcomes^(11,12)^. The WHO and Ethiopia national antenatal guidelines recommend providing nutritional counseling at every antenatal care (ANC) visit to improve dietary practices, nutrient intake and pregnancy outcomes^(1,13)^. These interventions, including Fe-folic acid supplementation and promoting pregnancy-specific foods^(14,15)^, are further supported in Ethiopia by the 2016 Blended Integrated Nutrition Learning Module (BINLM) packages to enhance healthcare providers’ ANC nutrition counseling skills^(16)^.

A pregnant woman’s understanding of proper nutrition is essential to meet increased dietary needs and achieve optimal health for both mother and fetus^(17)^. Evidence indicates that well-structured in-service training for antenatal care providers on counseling skills and pregnancy nutrition enhances their ability to educate pregnant women effectively, leading to improved maternal nutrition knowledge and dietary practices^(18,19)^. Despite recognising the importance of nutrition education, healthcare workers face barriers such as limited time, inadequate space, poor counseling skills and lack of documentation, resulting in insufficient nutrition education for women during ANC follow-ups^(20)^ and often provided only once, typically during the first ANC visit^(21)^.

Despite national guidelines recommended nutritional education and counseling at every ANC visits in Ethiopia, only 71 % of women receive it at least once^(10)^, indicating a significant gap in prenatal care for optimal nutrition. Furthermore, limited evidence on the extent and effectiveness of nutrition education and counselling on the knowledge and dietary practices of pregnant women using the BINLM in Addis Ababa, Ethiopia. Therefore, this study evaluated the effectiveness of facility-based nutrition education and counseling, delivered through the BINLM initiative, in improving the knowledge and dietary practices of pregnant women in Addis Ababa, Ethiopia.

## Materials and methods

### Study setting and period

The study was conducted in Addis Ababa, the capital city of Ethiopia from August 2017 to December 2017. Addis Ababa is stratified into 10 sub-cities with a total of 94 health centres, each sub-cities having 8–11 health centres^(22)^. In 2019, 97 % of pregnant women in Addis Ababa received ANC from skilled healthcare providers and 82 % of women had at least four ANC visits. However, only 19·4 % of women took Fe folate supplements for at least 90 d and 28·5 % did not take any supplementation throughout their pregnancy^(10)^.

### Study design

We conducted a cluster randomised controlled trial to evaluate the effect of nutrition education and counseling on the knowledge and dietary practice of pregnant women. Health centres served as clusters and a unit of randomisation. Each cluster was defined as a catchment area of a health centre, targeting the intervention at the cluster level, while outcomes were measured at the individual level (pregnant women attending ANC).

In the Ethiopian health system, each health centre serves a specific catchment population, and pregnant women in that area attend ANC at their respective health centres. We used a cluster-randomised design to minimize information contamination by following steps.

1. Excluded three non-governmental health centres and nine health centres with a monthly ANC caseload of fewer than fifty women.
2. Randomly select one health centre from each sub-city.
3. A matched pair for each randomly selected health centre was selected from the same sub-city ensuring non-adjacent catchment areas and health centres within ±15 % range of an average monthly ANC caseload from the first randomly selected health centre.

Data for the pair-matching purpose was obtained from the Addis Ababa Health Bureau (AAHB) database and sub-city Health Management Information System (HMIS) reports. Health centres were then randomised into either the intervention or control arm by an independent individual using a public container ballot. The twenty health centres were invited to participate before randomisation. Given the nature of the intervention, blinding was not possible for both healthcare providers and pregnant women. However, data collectors and supervisors remained blinded to the allocation.

### Study population

All pregnant women attending ANC follow-ups in Addis Ababa health centres during the study period were included in our study. We excluded pregnant women who were above 28 weeks of gestation at baseline, those with a history of chronic diseases (such as HIV, TB, diabetes, and hypertension), and those with multiple pregnancies. Healthcare providers are trained professionals, such as midwives, nurses and health officers in ANC units, who are providing essential services to support the health of pregnant women and their families. In Addis Ababa, health centres typically have four healthcare providers in their ANC units, all of whom were included in the study.

### Sample size determination

The sample size was calculated using a double population proportion formula for cluster randomised trials based on the dietary practice of the study. We hypothesised a 20 % improvement in dietary practice after the intervention among pregnant women in the intervention compared to the control arm. We assumed that 46 % of pregnant women already have good dietary practices during pregnancy and 53 % of pregnant women have knowledge of appropriate pregnancy nutrition before the intervention^(18)^.

The trial sample size was calculated based on the assumptions of an intracluster correlation coefficient of 0·04, 10 clusters per arm, a 10 % dropout rate, a power of 90 %, and a 95 % confidence level (CI). This resulted in a minimum required sample size of 330 pregnant women per arm. The sample size was calculated using Stata version 14 (StataCorp LP) statistical software.

The calculated sample size was then proportionally allocated to the health centres based on the average monthly ANC caseload. pregnant women were selected consecutively from the medical registry and followed up when they returned for their next ANC visit.

### Intervention

Nutrition education and counseling package was provided to ANC providers at the intervention centres, aimed at improving the knowledge and dietary practice of pregnant women. All healthcare providers working in ANC clinics of the intervention health centres received standardised in-service nutritional two-days training for using a module derived and modified from the BINLM, a national in-service nutrition training module prepared by the Federal Ministry of Health of Ethiopia^(16)^. The training was primarily facilitated by the principal investigator who is certified by training of trainers in BINLM^(23)^, with the support of research collaborators, Health Information Education and Communication experts and nutrition focal personnel from AAHB.

The first part of the training aimed at introducing pregnancy nutrition to ANC providers by addressing energy needs during pregnancy, pregnancy weight gain, food group classifications, food ingredients and lifestyle issues, food safety, common problems associated with pregnancy, benefits of fulfilling nutrient requirement during pregnancy and consequence of maternal malnutrition.

The second part of the training aimed at improving the communication skills of ANC providers. It covered nutrition education and counseling skills, how to analyse and deliver key messages and doable actions. The GALIDRA strategy (Greet, Ask, Listen, Identify, Discuss, Repeat, and Appoint) was used to counsel and reach an agreement with pregnant women and ANC providers. Additionally, the health belief model was employed to support message delivery and recommendations, helping to explain and predict health behaviours based on the perceived threats and benefits^(24,25)^. The details of this training package for ANC providers and its evaluation have been published previously^(26)^.

Thus, healthcare providers practiced providing simple, personalised and specific recommendations using case scenarios and role-plays. The training was supported by PowerPoint presentations, discussions and with additional materials such as summary pamphlets with personalised nutrition message for pregnant women.

The impact of the training on the knowledge and counseling skills of ANC providers was evaluated using a pre-post-evaluation question. Further discussions were held to address areas that need improvement. One-week supportive supervision was made to intervention arm health centres to help identify and solve any issues in implementing the training and filling any gaps.

Take-home brochures prepared in the local language (Amharic) were also distributed to pregnant women during their second ANC visits. The brochures include simple and easy-to-understand key messages and doable actions of maternal recommendations based on the health belief model, to reinforce the training and provide contentious support to pregnant women.

In this study, the control arm consisted of health facilities that continued providing standard care^(13)^ without receiving the nutrition education and counselling training, serving as a comparison group to assess the effectiveness of the intervention.

### Data collection procedure

Data were collected from all ANC providers and pregnant women at two time points. Initially, pre-intervention (baseline) data on the knowledge and dietary practices of pregnant women and the counseling skills of healthcare providers were collected. Interview questions were adapted from various sources including the FAO of the UN^(27)^, Food and Nutrition Technical Assistance^(27)^, FAO Guidelines for Assessing Nutrition-related Knowledge, Attitudes and Practices Manual and other relevant literature^(18,27,28)^. Interviews gather information on socio-demographic information (such as age, educational and occupational status, marital status and family size), obstetric information (e.g. parity, gravidity and gestational age), 24-h dietary diversity, seven days’ meal frequency and nutritional knowledge.

Then, end-line data were collected 20 weeks after baseline assessment using a similar module and procedure employed at baseline. We applied the same questionnaire used at the baseline except that one additional question was added at the end line. During the end line, we asked pregnant women if they received the specific take-home brochures, to assess any possible contamination that might have happened between intervention and control arms.

The data collection tool was first prepared in English then translated to Amharic and then back to English translation by another person to check for consistency. It was also pretested on a sample of pregnant women and healthcare providers from health centres that were not a part of the study. Questionnaires were modified after the pretest and data from the pretest was not included in the analysis of this study.

Trained health professionals conducted each round of data collection and supervision. Healthcare providers not working at ANC and delivery units within health centres collected the data from pregnant women, while health officers not affiliated with the selected health centres collected observation checklists.

### Follow-up protocol

After the baseline data collection, pregnant women were instructed to come for their next ANC follow-up before being interviewed at the end-line study. During the three consecutive antenatal care^(14)^ visits, healthcare providers delivered structured nutrition education and counseling sessions to the pregnant women. Contact information was collected from pregnant women at enrollment time to facilitate tracking. Women in the intervention arm received a take-home brochure after the baseline visit. Data collectors proactively contact women 3 d before their next ANC visit and again on the appointment day.

Lost to follow-up was declared when tracing the woman was unsuccessful, a pregnant woman moved outside the city during the study period, a woman changed health centers to a different arm, a woman was referred for various reasons and a woman explicitly declined further participation despite the data collector efforts.

### Outcome assessment and analysis

**Nutritional knowledge of pregnant women** was assessed by using six different constructed parameters, each analysed as a binary variable (Yes/No). A pregnant woman was considered knowledgeable if she knew that she needed to add one extra meal to her daily diet, use iodised salt for cooking, add salt when serving food, take Fe-folic acid supplements for at least 6 months, gain an appropriate amount of weight during pregnancy (10–14 kg) and initiate breast-feeding immediately after birth or within 1 hour.

The mean knowledge of women was calculated for questions regarding knowledge of food groups, components of a balanced diet, benefits of a balanced diet, consequence of undernutrition, use of Fe-folic acid supplements, issues to avoid/limit during pregnancy and solutions for common problems during pregnancy with the corresponding scores of 11, 8, 9, 8, 4, 7 and 5, respectively (Table 1).

**Table 1.** Definitions regarding nutritional knowledge of pregnant women along with their maximum score points

Knowledge outcomes	Definitions	Score points*
Food groups	Main sources of iron + vitamin A + protein + calcium	11
Component of a balanced diet	Meat poultry and fish + eggs + fruits + DGLV + dairy + pulses + grains + roots and tubers	8
Benefits of a balanced diet	Benefits of a balanced diet for a pregnant woman (5) + benefits of a balanced diet for the fetus (4)	9
Consequences of undernutrition	consequences of undernutrition for a pregnant woman (3) + consequences of undernutrition for the fetus (5)	8
Use of iron folic acid supplement	Cognitive development + prevent LBW + prevent birth defects + prevent anaemia	4
Things to avoid/limit	Alcohol + smoking + caffeine + raw food/milk + workload + stress + taking pills out of prescription	7
Common problems during pregnancy	Eat small and frequently + take IFA supplements after meals + drink more fluids + avoid sleeping immediately after meals + consult health care providers	5

DGLV, dark green leafy vegetables; DID, difference-in-differences.*Score points: – maximum possible scores.

**The dietary practice of pregnant women** was assessed using the qualitative 24-h recall method, where a list of ten food groups was used to calculate dietary diversity scores ranging from 1 to 10. Women with a score of 5 or higher were considered to have met the minimum dietary diversity, while those with a score below 5 were classified as having poor dietary diversity^(29)^. Similarly, dietary practices such as eating an extra meal, using iodised salt, adding salt when serving, gaining weight during pregnancy and following diet limitations were gathered and analysed as binary variables (Yes/No).

We calculated the seven-day meal frequency of pregnant women by asking them how many days they consumed each food group. Fe folic acid supplement, tea and coffee consumption were analysed using the previous 7 d’ frequency intake. Tea consumption was measured using the number of approximately 90 ml teacups per week, while coffee was measured using approximately 70 ml cups. We employed the mean difference (MD)-in-difference impact estimator to evaluate the intervention’s impact on food group consumption, Fe-folic acid supplements, tea and coffee.

### Statistical analysis

The data were entered, coded and cleaned using Epidata version 4.2.0 software and exported to Stata version 14.0 statistical software. The baseline characteristics of the women in both arms were summarised in a table with frequencies and proportions. Descriptive statistics (mean and sd) were calculated for all continuous variables, while frequency distribution was used for evaluating the distribution of categorical variables.

We used mixed-effect logistic regression to compare the baseline characteristics of pregnant women who lost follow-up and who completed the study. Similarly, baseline comparisons of healthcare providers were performed using mixed-effect logistic regression.

Mixed-effect linear regression analysis was employed to analyse the primary and secondary outcomes, with the health centre catchment area as a random effect to adjusted the clustering effect to get robust standard errors. The results of all outcome variables are presented as the difference in difference (DID) impact estimators to display the net effect of the intervention. Differences in proportions (DP) (end-line result – baseline result of that outcome) are also presented along with the DID estimates. Statistical significance was declared at *P* value < 0·05 for all outcomes. All knowledge and dietary practice outcomes were treated separately in a mixed-effect regression model. All *P* values in each table (Tables 2–5) were adjusted for multiplicity issues using Finner’s adjusted test^(30)^, after the mixed effect regression analysis, and adjustment was performed using WinPepi software version 11.65.

**Table 2.** Baseline characteristics of pregnant women by study arm from the selected health centres in Addis Ababa, Ethiopia, 2017

Characteristics	Intervention *n* (347)		Control *n* (336)	
	Frequency	%	Frequency	%
Age (in completed years)				
18–24	155	44·7	134	39·9
25–34	176	50·7	183	54·5
35–45	16	4·6	19	5·6
Religion				
Orthodox	249	71·8	242	72·0
Muslim	68	19·6	73	21·7
Protestant	30	8·6	21	6·2
Ethnicity				
Amhara	162	46·7	168	50·00
Tigre	21	6·1	11	3·3
Oromo	71	20·5	64	19·1
Gurage	79	22·8	74	22·0
Silt’e	14	4·0	19	5·6
Educational status of a woman				
Can’t read and write	40	11·5	50	14·9
Can read and write	63	18·2	66	19·6
Primary school	122	35·2	106	31·5
Secondary school	62	17·9	60	17·9
College and above	60	17·3	54	16·1
Occupational status of a woman				
Unemployed	50	14·4	43	12·8
Student	12	3·5	4	1·2
Housewife	119	34·3	130	38·7
Daily labourer	11	3·2	8	2·4
Merchant	13	3·7	9	2·7
Civil servant	92	26·5	80	23·8
Self-employed	50	14·4	62	18·4
Marital status				
Single	13	3·7	5	1·5
Divorced/separated	3	0·9	4	1·2
Married/living together	331	95·4	327	97·3
Husband’s educational status				
Can’t read and write	18	5·4	21	6·4
Can read and write	54	16·2	70	21·4
Primary school	87	26·1	86	26·3
Secondary school	85	25·5	65	19·9
College and above	89	26·7	85	25·9
Husband’s occupational status (main income)				
Unemployed	4	1·2	4	1·2
Daily labourer	27	8·1	24	7·3
Merchant	36	10·8	22	6·7
Civil servant	144	43·2	122	37·3
Self-employed	122	36·6	155	47·4
Family size				
<= 2	195	56·1	171	50·9
3–4	82	23·6	96	28·6
> 4	70	20·2	69	20·5
Family monthly income (in birr)				
< 2000	41	11·82	34	10·12
2000–2999	52	14·99	45	13·39
3000–4499	73	21·04	75	22·32
>= 4500	67	19·31	47	13·99
Missing	114	32·85	135	40·18
Gestational age (in weeks)				
0–16	145	41·8	137	40·8
17–28	202	58·2	199	59·2
Gravidity				
Prim gravida	186	53·6	158	47·0
Multi gravida	161	46·4	178	52·9
Parity				
Nulliparous	211	60·8	181	53·9
Prim parous	85	24·5	104	30·9
Multiparous	51	14·7	51	15·2
Number of ANC visits prior to enrollment				
First	212	61·1	186	55·4
Second	135	38·9	150	44·6

**Table 3.** Nutritional knowledge of pregnant women by study arm and study round (baseline and end line) from the selected health centres in Addis Ababa, 2017

Knowledge outcomes		Intervention^‡^		Control^§^		DID impact estimator^||^		
		*n*	%	*n*	%	%	95 % CI^¶^	ICC**
One additional meal	Baseline*	121	34·9	124	36·9	49·9****	37·82, 62·16	0·04
	Follow-up^†^	232	89·2	99	41·2			
Using iodised salt	Baseline*	256	73·8	263	78·3	23·0****	18·39, 27·39	0·04
	Follow-up^†^	255	98·1	192	80·0			
Adding salt when serving instead of cooking	Baseline*	27	7·8	31	9·2	77·4****	70·67, 84·13	0·01
	Follow-up^†^	228	87·7	29	12·1			
Duration of IFA supplement^††^	Baseline*	25	7·2	28	8·3	68·0****	57·36, 79·09	0·03
	Follow-up^†^	203	78·1	28	11·7			
Pregnancy weight gain	Baseline*	70	20·2	60	17·9	50·6****	43·06, 58·11	0·03
	Follow-up^†^	201	77·3	59	24·6			
Early initiation of breast-feeding	Baseline*	278	80·1	260	77·4	9·3***	−1·43, 20·13	0·03
	Follow-up^†^	242	93·1	193	80·4			

*Baseline *n* 683, ^†^follow-up *n* 500, ^‡^baseline intervention *n* 347, follow-up intervention *n* 260, ^§^baseline control *n* 336, follow-up control *n* 240, ^||^difference in difference impact estimator using mixed-effect linear regression with health centre catchment area as random effects adjusted for women’s marital status, gravidity, number of visits, women occupational status and husband’s occupational status, ^¶^adjusted for clustering effect to get a robust se, CI, **ICC, intracluster correlation coefficient, ^††^IFA – iron-folic acid supplement, ****P < 0·01,***P > 0·05. All *P* values in this table were adjusted for multiplicity issues using Finner’s adjusted test after the mixed-effect regression.

DID, difference-in-differences.

**Table 4.** Mean nutritional knowledge of pregnant women by study arm and study round (baseline and end line) of selected health centres in Addis Ababa, 2017

Knowledge outcomes		Intervention^‡^		Control^§^		DID impact estimator^||^	95 % CI^¶^
		Mean	sd**	Mean	sd**	Mean score	
Food groups							
Out of 11 points	Baseline*	1·9	1·5	2·0	1·6	3·4***	2·88, 4·0
	Follow-up^†^	5·4	2·1	2·0	1·5		
Component of a balanced diet							
Out of 8 points	Baseline*	2·5	1·2	2·5	1·2	2·1***	1·7, 2·43
	Follow-up^†^	4·9	1·4	2·9	1·1		
Benefits of a balanced diet							
Out of 9 points	Baseline*	2·2	0·9	2·2	1·0	2·0***	1·49, 2·51
	Follow-up^†^	4·3	1·5	2·3	0·9		
Consequences of undernutrition							
Out of 8 points	Baseline*	2·1	0·9	2·1	0·9	2·2***	1·89, 2·53
	Follow-up^†^	4·4	1·3	2·2	0·9		
Use of iron folic acid supplement							
Out of 4 points	Baseline*	0·6	0·6	0·6	0·6	1·4***	1·16, 1·55
	Follow-up^†^	2·0	0·9	0·7	0·6		
What to avoid/limit during pregnancy							
Out of 7 points	Baseline*	1·8	1·2	1·9	1·1	2·1***	1·68, 2·48
	Follow-up^†^	3·9	1·1	2·0	0·9		
Solutions for common problems during pregnancy							
Out of 5 points	Baseline*	1·5	0·7	1·6	0·7	1·3***	1·05, 1·63
	Follow-up^†^	3·0	0·9	1·8	0·6		

DID, difference-in-differences.

Number of clusters = 20, *baseline *n* 683, ^†^follow-up *n* 500, ^‡^baseline intervention *n* 347, follow-up intervention *n* 260, ^§^baseline control *n* 336, follow-up control *n* 240, ^||^average difference in difference impact estimator using mixed-effect linear regression with health centre catchment area as random-effects adjusted for women’s marital status, gravidity, number of visits, women occupational status and husband’s occupational status, ^¶^Adjusted for clustering effect to get robust se, CI, **sd, ***P < 0·01. All *P* values in this table were adjusted for multiplicity issue using Finner’s adjusted test after the mixed-effect regression.

**Table 5.** Dietary practice of pregnant women by study arm and study round (baseline and end line) in selected health centres in Addis Ababa, 2017

Outcomes		Intervention^‡^		Control^§^		DID impact estimator^||^	95 % CI^¶^	ICC**
		*n*	%	*n*	%	%		
Dietary diversity	Baseline*	189	54·5	195	58·0	32·3***	18·07, 46·59	0·05
	Follow-up^†^	243	93·5	150	62·5			
Had one additional meal	Baseline*	73	21·0	85	25·3	12·8***	3·58, 22·08	0·01
	Follow-up^†^	150	57·7	120	50·0			
Used iodized salt	Baseline*	220	63·4	230	68·4	36·2***	29·69, 42·61	0·07
	Follow-up^†^	254	97·7	158	65·8			
Add salt when serving	Baseline*	8	2·3	5	1·5	73·4***	64·37, 82·47	0·02
	Follow-up^†^	201	77·3	9	3·7			
Follow weight during pregnancy	Baseline*	167	48·1	155	46·1	32·1***	19·03, 45·19	0·01
	Follow-up^†^	230	88·5	127	52·9			
Avoid diet restrictions on healthy foods during pregnancy	Baseline*	23	6·6	18	5·4	−3·2	−6·64, 0·16	0·02
	Follow-up^†^	3	1·1	11	4·6			

DID, difference-in-differences.

Number of clusters = 20, *baseline *n* 683, ^†^follow-up *n* 500, ^‡^baseline intervention *n* 347, follow-up intervention *n* 260, ^§^baseline control *n* 336, follow-up control *n* 240, ^||^difference in difference impact estimator using mixed-effect linear regression with health centre catchment area as random effects adjusted for women’s marital status, gravidity, number of visits, women occupational status and husband’s occupational status, ^¶^Adjusted for clustering effect to get a robust se, CI, **ICC, intracluster correlation coefficient, ***P < 0·01. All *P* values in this table were adjusted for multiplicity issues using Finner’s adjusted test after the mixed-effect regression.

## Results

**Figure 1.**
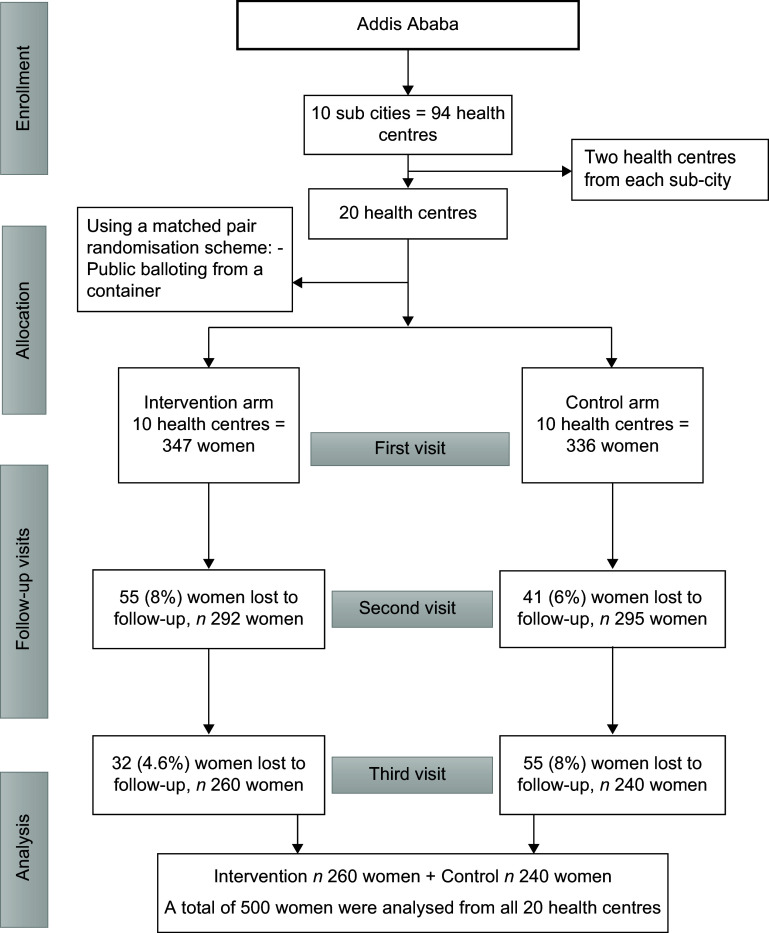
The flow diagram illustrating the distribution of health centres, pregnant women’s enrollment and allocation in each arm and analytic sample. A total of 683 pregnant women were enrolled in the study. After the first ANC visit, 96 (14 %) women were lost (55 intervention, 41 control). At the third visit, 87 (12 %) more were lost (32 intervention, 55 control). Overall, there were 183 (26·6 %) losses to follow-up among the 20 health centres. ANC, antenatal care.

Out of the 683 women enrolled at baseline from twenty health centres, ninety-six (14 %) were lost before the second ANC visit and 87 (12 %) during the third visit (Figure 1).

Table 2 presents the comparison of the baseline characteristics between the intervention and control arms. The overall baseline characteristics were similar between the two arms, indicating proper randomisation of the trial. All pregnant women in the intervention arm who attended their second visit (*n* 292) received the take-home brochure. In contrast, none of the pregnant women in the control arm reported having seen or received the brochure (Table 2).

### Nutritional knowledge of pregnant women

Pregnant women in the intervention arm had a significantly higher knowledge on adding salt when serving (DP intervention *v*. control, (79·9 % *v*. 2·9 %); DID 77·4 %; 95 % CI: 70·7, 84·1), knowledge on using iodised salt (24·3 % *v*. 1·7 %; DID 23 %; 95 % CI: 18·4, 27·4) and knowledge on duration of iron-folic acid supplementation (70·9 % *v*. 3·4 %; DID 68 %; 95 % CI: 57·4, 79·1). A significantly higher knowledge was found among pregnant women in the intervention arm on having one additional meal (54·3 % *v*. 4·3 %; DID 49·9 %; 95 % CI: 37·8, 62·2) and pregnancy weight gain (57·1 % *v*. 6·7 %; DID 50·6 %; 95 % CI: 43·1, 58·1). However, there was no significant difference in knowledge on the initiation of breast-feeding between the intervention and control arms (DP 13·0 % *v*. 3·0 %; DID 9·3 %; 95 % CI: –1·4, 20·1) (Table 3).

Table 3 presents the mean nutritional knowledge of pregnant women by study arm. Pregnant women in the intervention arm showed a significant improvement in knowledge about food groups, use of IFA supplements, benefits of a balanced diet and consequences of undernutrition. The intervention arm showed a significant improvement in knowledge of the solutions for common pregnancy concerns such as nausea, vomiting, heartburn and constipation (Table 4).

### Dietary practice of pregnant women

We found that dietary diversity was significantly improved among pregnant women in the intervention arm (DP 39·0 % *v*. 4·5 %; DID 32·3 %; 95 % CI: 18·1, 46·6). A significant improvement was also observed among women of intervention arm on the use of iodised salt (DP 34·3 % *v*. 2·6 %; DID 36·2 %; 95 % CI: 29·7, 42·6), adding salt when serving food (DP 75·0 % *v*. 2·2 %; DID 73·4 %; 95 % CI: 64·4, 82·5), adding one additional meal to their diet (DP 36·7 % *v*. 24·7 %; DID 12·8 %; 95 % CI: 3·6, 22·1) and monitoring their weight during pregnancy (DP 40·4 % *v*. 6·8 %; DID 32·1 %; 95 % CI: 19·0, 45·2). However, we did not find a significant difference in avoiding diet restriction of healthy foods during pregnancy (DP –5·5 % *v*. 0·8 %; DID –3·2 %; 95 % CI: –6·6, 0·16) (Table 5).

Pregnant women in the intervention arm increased their dairy consumption by one more day per week (MD 1·2 *v*. –0·1; DID 1·2; 95 % CI: 0·7, 1·8). In the intervention arm, there was a significant improvement in IFA intake by three more days per week compared to the control arm (MD 4·9 *v*. 1·6; DID 3·2; 95 % CI: 2·1, 4·3). There was also a significant reduction in tea consumption (MD –3·9 *v*. –1·1; DID –4·1; 95 % CI: –4·99, –3·13) and coffee consumption (MD –4·2 *v*. –0·5; DID –5·1; 95 % CI: –6·67, –3·56) in the intervention arm. However, there was no significant difference in consumption of grains (MD 0·5 *v*. 0·6; DID –0·19; 95 % CI: –0·04, 1·28), vitamin A-rich fruits and vegetables (MD 0·8 *v*. –0·3; DID 0·53; 95 % CI: 0·04, 1·44), meat and poultry (MD 0·8 *v*. –0·3; DID 0·7; 95 % CI: 0·11, 1·3) and fish (MD 0·2 *v*. 0·0; DID 0·25; 95 % CI: 0·07, 0·42) between the two arms (Table 6).

**Table 6.** Seven days’ food intake, iron supplementation and caffeine intake among pregnant women, in Addis Ababa, 2017

Outcomes		Intervention^‡^		Control^§^		DID impact estimator^||^	95 % CI^¶^
		Mean	sd	Mean	sd	Mean score	
Grains	Baseline*	6·0	1·7	6·0	1·8	−0·19	−0·04, 1·28
	Follow-up^†^	6·5	1·0	6·6	0·9		
Dairy	Baseline*	2·2	2·3	2·1	2·3	1·2****	0·65, 1·82
	Follow-up^†^	3·4	2·0	2·0	1·7		
Meat and poultry	Baseline*	1·8	1·5	2·0	1·5	0·7	0·11, 1·27
	Follow-up^†^	2·6	1·4	1·7	1·2		
Fish	Baseline*	0·1	0·3	0·1	0·3	0·25	0·07, 0·42
	Follow-up^†^	0·3	0·7	0·1	0·3		
Vitamin A-rich fruits and vegetables	Baseline*	2·0	1·9	2·0	1·9	0·53	0·55, 1·44
	Follow-up^†^	2·8	1·8	1·7	1·4		
Iron folic acid supplementation^††^	Baseline*	1·4	2·7	1·4	2·6	3·2****	2·14, 4·26
	Follow-up^†^	6·3	1·9	3·0	3·1		
Tea	Baseline*	6·3	5·9	6·7	5·7	−4·1****	−4·99, –3·13
	Follow-up^†^	2·4	3·4	5·6	5·9		
Coffee	Baseline*	5·7	6·8	5·2	6·5	−5·1***	−6·67, –3·56
	Follow-up^†^	1·5	3·1	4·7	6·4		

DID, difference-in-differences.

Number of clusters = 20 *baseline *n* 683, ^†^follow-up *n* 500, ^‡^baseline intervention *n* 347, follow-up intervention *n* 260, ^§^baseline control *n* 336, follow-up control *n* 240, ^||^average difference in difference impact estimator using mixed-effect linear regression with health centre catchment area as random effects adjusted for women’s marital status, gravidity, number of visits, women occupational status and husband’s occupational status, ^¶^Adjust for clustering effect to get a robust se, CI, **sd – standard deviation, ***P < 0·001, ****P < 0·01, ^††^analysis of iron folic acid supplement is adjusted for the number of antenatal care visits. All *P* values in this table were adjusted for multiplicity issues using Finner’s adjusted test after the mixed-effect regression.

## Discussion

In the present study, facility-based nutrition education and counseling packages, along with the distribution of take-home brochures, were found to improve the overall nutrition knowledge and dietary practice of pregnant women. Women in the intervention arm showed significant improvements in nutrition knowledge and dietary practices than control arm, highlighting the value of integrated training, resources, and mentorship in enhancing ANC quality and maternal health. Consistent with findings from a study conducted in the East Showa Zone, Ethiopia, nutrition education and counseling utilising the BINLM module demonstrated an improvement in the nutritional knowledge of pregnant women^(31)^.

Previous studies have shown that nutrition education and counseling interventions increase pregnant women’s knowledge of nutrition and dietary practices^(18,19,32)^. These studies showed that maternal knowledge, recall of nutritional recommendations and dietary practices can be improved with education from skilled healthcare providers. Similar evidence has indicated that training of healthcare providers in nutrition and counseling effectively increases maternal recall of nutritional recommendations for child feeding, with significant improvements observed both immediately and 180 d post-intervention^(33,34)^.

After the intervention, the weekly consumption of caffeine was reduced by four cups of tea and five cups of coffee. However, pregnant women continued to consume tea and coffee within the WHO recommendation limits of up to one cup of coffee and two cups of tea per day^(1)^.

The finding of this study highlighted that pregnant women in the intervention arm had significantly higher adherence to IFA supplementation compared with the control group. Consistent with other studies showing increased IFA coverage with nutrition education^(35,36)^, this highlights the need to enhance ANC services with comprehensive nutrition education and counseling for all pregnant women to improve maternal and child health services.

Our nutritional intervention package included easy-to-understand take-home brochures, designed using the health belief model and contained simple doable actions and maternal recommendations for pregnant women. Research also supported the effectiveness of applying behavioural change theories to health and nutrition education in achieving the desired behavioural changes that lead to positive outcomes^(24,37)^.

Generally, the intervention group demonstrated significantly greater improvements in dietary practices compared with the control group, which aligned with their enhanced nutritional knowledge. This suggests that well-designed nutrition education and counseling can effectively influence both knowledge and behaviour. Furthermore, the findings highlight the importance of not only the content but also the mode of communication in delivering nutritional information. However, we found that our intervention has no significant effect on pregnant women’s knowledge of early initiation of breast-feeding. Similar findings were also observed in Pelotas, Brazil, where maternal recall of early initiation of breast-feeding was found to be insignificant^(34)^.

In this study, we found no significant improvement in seven days’ consumption of grains, meat and poultry and fish. This could be due to the high cost of meat and low availability of fish in Ethiopia, leading to limited consumption of animal-based products by most pregnant women^(38)^.

In addition to improvements in knowledge and dietary practices, personalised nutrition education and counseling might relate to the decreased lost of follow-up in the second visit among pregnant women of the intervention compared to the control arm. It is evident from health management information system reports of ANC units in Ethiopia, particularly in Addis Ababa that ANC visit adherence decreases as the number of visits increases^(39)^. Nevertheless, pregnant women in our study might have shown interest in their next follow-up visits because of the improved and personalised approach of healthcare providers and the easily understandability of recommended doable actions. Thus, the increased adherence to third and fourth visits in our study might pertain to it. In WHO ANC guidelines, it is noted that irrespective of the number of recommended contacts in the ANC model, women will not attend ANC if the quality of ANC is poor and women’s experience of it is negative^(40)^.

Our intervention might yield a different result in dietary practice among pregnant women living in rural parts of Ethiopia, where the setting, socio-economic status, availability and cost of food are different from those in the capital city, Addis Ababa. In addition, pregnant women in Addis Ababa might be more receptive to changes in practice due to the availability of different sources of information. Yet, nutrition education and counseling are provided in the usual public health services available to all pregnant women living all over the country, the knowledge of recommendations might not differ in other settings.

The strength of our study includes blinding data collectors and supervisors, incorporating behavioural models into our intervention and collecting baseline data before implementation of intervention to estimate the actual impact.

The study exposed the potential for socially desirable answers from pregnant women and recall biases in weekly dietary assessment questions, which may have overestimated the impact of nutrition education counseling on dietary practice and knowledge of pregnant women. However, data collectors were given training, and data collection was done the same way between the two arms.

The proportion of women lost to follow-up exceeded our initial expectations. However, the distribution of lost-to-follow-up cases did not differ significantly between the two study arms. Upon recalculating the study’s power at the end of the study, it was determined to be 87 %. Therefore, we wish to disclose that, instead of the initially assumed 90 % power, our study achieved 87 % power at the end due to the reduced sample size resulting from lost-to-follow-up cases.

Facility-based nutrition education and counseling, supported by the BINLM package and delivered by trained healthcare providers with take-home brochures, could enhance pregnant women’s nutritional knowledge and dietary diversity. We recommend that policymakers implement such nutrition education through trained providers using the BINLM package and develop antenatal care guidelines that incorporate take-home brochures with key messages and actionable steps. Additionally, updated ANC guidelines should explicitly recommend and facilitate the use of take-home brochures that convey vital health messages and practical guidance. The programme should be scaled up to other cities, particularly rural areas, to enhance the national dietary intake. Additionally, further studies should assess the impact of the BINLM package on pregnancy outcomes, weight gain, anthropometry and the effectiveness of incorporating audio-visual messages. This could involve using quantitative dietary assessments and replicating the study in rural settings.
